# How accurate is image guided radiation therapy (IGRT) delivered with a micro-irradiator?

**DOI:** 10.1088/1742-6596/444/1/012070

**Published:** 2013

**Authors:** M Oldham, J Newton, L Rankine, J Adamovics, D Kirsch, S Das

**Affiliations:** 1 Department of Radiation Oncology, Durham, USA; 2 Duke University Medical Center, Durham, USA; 3 Rider University, NJ, USA

## Abstract

There is significant interest in delivering precisely targeted small-volume radiation treatments, in the pre-clinical setting, to study dose-volume relationships with tumor control and normal tissue damage. In this work we investigate the IGRT targeting accuracy of the XRad225Cx system from Precision x-Ray using high resolution 3D dosimetry techniques. Initial results revealed a significant targeting error of about 2.4mm. This error was reduced to within 0.5mm after the IGRT hardware and software had been recalibrated. The facility for 3D dosimetry was essential to gain a comprehensive understanding of the targeting error in 3D.

## 1. Introduction

We recently commissioned the treatment beams from a small field biological irradiator, the XRad225Cx from Precision x-Ray, for pre-clinical applications in our department [1, 2]. The XRad225 can produce a variety of small circular or square 225kVp photon beams ranging in size from 1mm to 40mm in maximum dimension. Initial commissioning focused on characterizing the radiation beams in terms of percent-depth-dose (PDD), two-dimensional (2D) profiles at various depths, and output factors. In the present work we extend this effort to evaluate the accuracy and capability of the cone-beam-CT (CBCT) image-guided radiation therapy (IGRT) capability of the system.

## 2. Methods

The accuracy of IGRT treatments was evaluated on Presage 3D dosimeters (5cm diameter cylinders of 5cm length), each containing 5 physical target points dispersed within the dosimeter. The target points were created by drilling channels (1.5mm diameter) of varying depth into the Presage dosimeter, parallel to the cylinder axis, but spatially offset. The end of each drill channel tapered to a sharp point, corresponding to a target point. The Presage cylinder was taped horizontally onto the XRad225 treatment stage to simulate a rat treatment. CBCT IGRT was then performed to position the tip of each drill-channel (the target point) at the isocenter. Each target point was treated with a 7 field equiangular coplanar treatment delivered with the small 2.5mm cone. Each beam delivered about 1Gy to the isocenter, leading to a total isocentric dose of about 7Gy. Immediately after irradiation of all targets, the Presage dosimeter was imaged in the DLOS optical-CT scanner [3]. The DLOS system produced isotropic 0.5mm data throughout the Presage cylinder. Post-irradiation scans were corrected by pre-irradiation scans acquired the day before treatment. In both scans the drill channels in the Presage dosimeter were filled with refractive matching fluid, to minimize refraction artifacts around the channels. Scanning involved acquiring 720 projections over 360 degrees, which took about 20minutes.

The 0.5mm isotropic 3D images returned from the DLOS scanner report the change in optical-density (OD) throughout the Presage dosimeter, which is well established to be proportional to absorbed dose [4]. The OD distribution corresponds to the relative dose distribution throughout the dosimeter. The accuracy of the IGRT treatments in 3D was then determined by studying positional discrepancies between the target points and the intersection point of the corresponding treatment beams. The experiment was repeated on two more Presage dosimeters using 1mm and 5mm cones for delivery. All other dosimeter parameters, prescribed dose and irradiation methods were kept constant. This provided the grounds for a statistical analysis on IGRT targeting accuracy

## 3. Results

**Figure 1:**
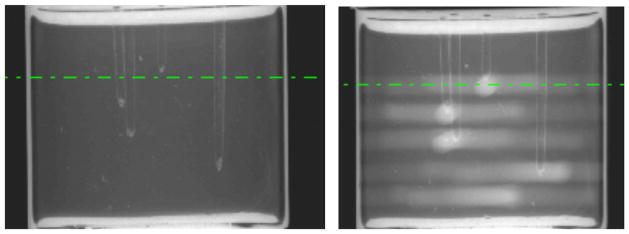
Illustrative projection images through (a) an un-irradiated, and (b) an irradiated Presage dosimeter.

Single projection images through an arbitrary view through the Presage dosimeter before and after irradiation is shown in figure 1. The four target points are visible in figure 1a, as the tapered ends of the vertical drill-channels. Figure 1b shows the same projection after irradiation, where now the coplanar beam tracks and intersection point of each treatment are visible.

**Figure 2:**
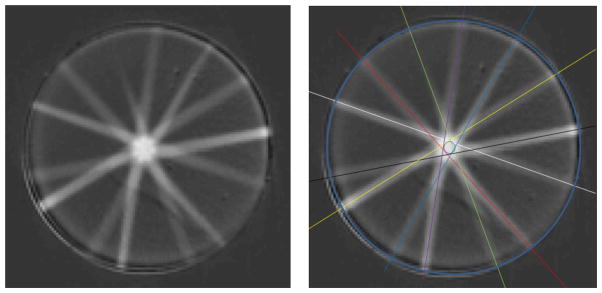
(a) an axial view through the Presage dosimeter at a plane through one of the 4 target points. (b) the same image but with central beam line annotations to highlight the cone misalignment.

Axial views through the results obtained on the initial run of this experiment are shown in figure 2. A misalignment is apparent because the center lines of each beam do not intersect at the same point. For our analysis, we defined *cone misalignment* as the radius of the smallest circle drawn at the isocenter which intersects all beams. *Targeting accuracy* was defined as the distance from mechanical isocenter to the tip of drilled holes. This misalignment was consistent for all 4 target points.

**Figure 3:**
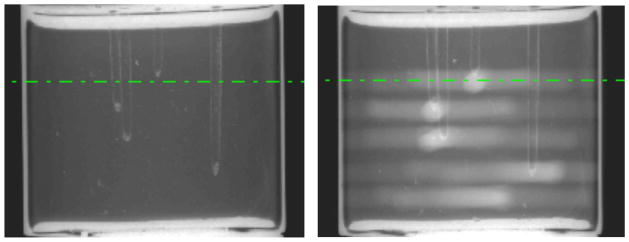
Illustrative projection images, after recalibration of the IGRT system, through (a) an un-irradiated, and (b) an irradiated Presage dosimeter.

**Figure 4:**
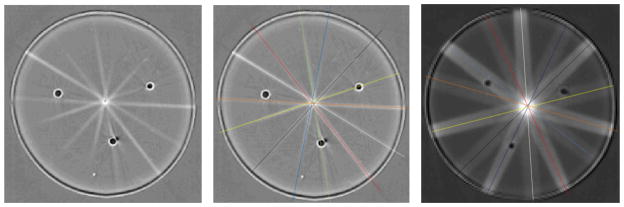
(a) axial view through the Presage dosimeter at a plane through one of the 4 target points treated with a 1mm cone. (b) the same image but with central beam line annotations to highlight much improved alignment compared with Figure 2. (c) similar image through another target point treated with a 5mm cone.

A statistical analysis averaging the discrepancies over all four target points found an average cone misalignment of 2.4±0.6mm and targeting error of 2.1±0.6mm (see Table 1). This was substantially greater than the nominal advertised system accuracy of 0.5mm. The system and online targeting software was re-calibrated. The experiment was repeated on three more Presage dosimeters also containing the 4 target drill channels. Delivery of the 7 beam coplanar irradiation was performed using cone sizes of 2.5mm, 1mm, and 5mm. Results from this second experiment are shown in figure 3 and 4.

Statistical analysis averaging the discrepancies over all four target points after the IGRT system had been re-calibrated was performed for each of the three dosimeters and recorded in Table 1. The cone misalignment and targeting accuracy of these three dosimeters provided the means for further statistical analysis. Results indicate that after recalibration the cone misalignment was <*r*> = 0.5mm (σ_<_*_r_*_>_ = 0.3mm) and targeting accuracy was <*dr*> = 0.5mm (σ_<_*_dr_*_>_ = 0.4mm), which is in line with the nominal advertised system accuracy of 0.5mm.

## 4. Conclusions

The DLOS/Presage 3D dosimetry system enabled rigorous evaluation of the IGRT targeting accuracy of the XRad225 system. The ability to image with isotropic 0.5mm resolution (0.3mm for cone size 1mm) was vital for understanding the true accuracy of the system, especially for very small fields like the 1mm diameter beams shown in figure 4.

## Figures and Tables

**Table 1:** Analysis of the cone misalignment and targeting accuracy for the four dosimeters. The xy-plane was defined as the plane traversed by the radiation beams (e.g. figure. 4). The z-axis was the longitudinal direction, normal to the axial xy-plane. Each entry shows the average over 4 target points and one standard deviation in parentheses

Cone Size [mm]	Recon. px size [mm]	Cone Misalignment [mm] Average (1.s.d.)			Targeting Accuracy [mm] Average (1.s.d.)			
		*xy*-plane	z*	*r*	*dx*	*dy*	dz*	*dr*
2.5	0.5	1.3 (0.1)	2.1 (0.6)	**2.4** (0.6)	0.05 (0.1)	0.08 (0.04)	2.1 (0.6)	**2.1** (0.6)
2.5	0.5	0.18 (0.04)	0.4 (0.1)	**0.5** (0.1)	0.07 (0.2)	−0.09 (0.2)	0.4 (0.1)	**0.5** (0.3)

* In the *z*-direction, *Cone Misalignment* and *Targeting Accuracy* were obtained from the same measurement.
